# Translation and Linguistic Validation of BIS (Body Image Scale) for Breast Cancer Patients in India

**DOI:** 10.1007/s13193-024-02037-2

**Published:** 2024-08-14

**Authors:** Preeti Belani, Tabassum Wadasadawala, Rajiv Sarin, Rima Pathak, Revathy Krishnamurthy, Naseera Syeda, Sonal Chavan

**Affiliations:** Department of Radiation Oncology, Breast DMG (Disease Management Group), Tata Memorial Centre, Homi Bhabha National Institute, Dr Ernest Borges Road, Parel, Navi Mumbai 400012 India

**Keywords:** PROMS (patient-reported outcome measures), BIS (body image scale), EORTC BR-23 (Breast Cancer), Breast cancer, QOL (quality of life)

## Abstract

A number of PROMs (patient-reported outcome measures) have been developed and validated for English-speaking populations but with limited utility in non-English-speaking countries. Body image scale (BIS) is a type of PROM which assesses the changes in the body image of a patient diagnosed with cancer. The current study was carried out to translate the BIS in Hindi and Marathi. Out of the 10 questions in the BIS questionnaire, Hindi and Marathi translations were already available for 4 questions (Questions no. 2, 4, 5, and 9) from the EORTC BR23 questionnaire. Remaining questions were translated using the EORTC translation manual, which includes forward translation, reconciliation, backward translation, proofreading, and pilot testing. Pilot testing done on 10 patients each for Hindi and Marathi showed at least 80% acceptability for all the questions for both Hindi and Marathi translations. Both the translated versions will be used for validation in a large cohort of breast cancer patients. The Hindi and Marathi versions of the BIS have very good acceptability and can be utilized for clinical trials once validated in cancer patients.

**Supplementary Information:**

The online version contains supplementary material available at 10.1007/s13193-024-02037-2.

## Background

Treatment for breast cancer involves multidisciplinary care. Management algorithms include surgery, chemotherapy, radiation therapy, targeted therapy, and endocrine therapy. Adjuvant radiotherapy following breast-conserving surgery is the current standard of care for early-stage breast cancers, as the survival and local control rates have been shown to be equal to mastectomy [1, 2]. With the improvement in the efficacy of the neoadjuvant therapies for locally advanced breast cancer, higher response rates are achieved that confer an excellent opportunity for breast conservation. Hence, more and more women are now undergoing breast conservation [3, 4].

Breast cancer has implications beyond the physical disease and the aftermath of treatment complications often leads to considerable psychological and emotional distress. The treatment, even if it is lifesaving, often creates morbidity of its own. Cosmetic outcome is considered one of the paramount endpoints to measure the success of breast conservation. To achieve an optimal cosmetic outcome, oncoplastic breast surgery (OPBS) is being increasingly adopted. Improved surgical techniques in the form of OPBS provide superior cosmetic outcomes as compared to traditional breast conservation surgery while offering comparable oncological safety. It allows for excision of larger volumes of breast tissue and extends the option of breast conservation to more patients with larger tumors, multifocal disease, and extensive intra-ductal component [5].

For anyone having battled and survived cancer, quality of life (QOL) post-treatment becomes extremely important. Assessing the quality of life in patients and cancer survivors was earlier restricted to research settings. However, recently, it is slowly seen moving to routine clinical practice and is commonly referred to as “patient-reported outcomes (PRO)”. These are defined as any report of the status of a patient’s health condition that comes directly from the patient without interpretation of the patient’s response by a clinician or anyone else. PROMs (patient-reported outcomes measures) are instruments that are used to measure the PROs and are most often self-report questionnaires [6].

A number of PROMs have been developed and validated for English-speaking populations [7]. However, their utility in non-English-speaking countries is limited. Hence, attempts are commonly made to validate such patient-reported outcome instruments to other languages to increase their applicability [8–10].

For this purpose, an instrument has to be systematically translated into a native language. Such a translation must be comparable to the original, accurate in collecting the intended data, easy to understand to a generic reader, and, most importantly, respect the cultural differences that come naturally as the geographical location of the target population changes. There are various factors that must be considered in such translations, including the cultural differences between the concerned populations, lifestyle, and acceptable social practices [11, 12].

Such a cross-cultural, language translation is a complicated process. It is possible that in-spite of having the same meaning, changing of context changes the implication of a word in one language, not in another. For example, the Hindi word “takleef” (तकलीफ़) is commonly used for discomfort related to a physical activity or an emotionally distressing situation; however, in English, the word “discomfort,” which is a literal translation of the word “takleef” usually corresponds to mostly physical discomfort.

The need for appropriately translated and validated PROMs is ever-increasing now with multinational clinical trials in place. Especially for breast cancer, the treatment modalities are rapidly changing, with universal implications, generating a need for PROMs in various regional languages. There is an obvious need for outcome-measuring instruments that are uniform and applicable in various cultural settings by virtue of their translation. To address this requirement, Beaton et al. have published guidelines for the process of cross-cultural adaptation of self-report measures [13]. EORTC and MAPI guidelines are also available for standardized translations of various questionnaires and PROMs [14, 15].

Body image scale (BIS) is a type of PROM questionnaire which assesses the changes in the body image of a patient diagnosed with cancer, on a 4-point scale that ranges from “not at all” to “very much.” It was constructed in collaboration with EORTC and tested on British cancer patients. The scale showed high reliability (Cronbach’s alpha 0.93) and good clinical validity [16].

BIS has been translated into various languages. Shunmugasundaram et al. translated and validated BIS in three Indian languages (Hindi, Tamil, and Telugu) and validated in head and neck cancer patients [17, 18]. The current study was carried out to translate the BIS in Hindi and Marathi, to be later validated in a large cohort of breast cancer patients.

## Materials and Methods

The Hindi or Marathi translations for the BIS were not available at the time of initiation of the study. Hence, the EORTC manual for linguistic translation was followed, and BIS was translated into both languages. Out of the 10 questions in the BIS questionnaire, Hindi and Marathi translations were already available for 4 questions (Questions no. 2, 4, 5, and 9) that constitute the body image scale (Q 39 to Q 42) of the EORTC breast cancer–specific instrument, i.e., BR-23 as shown in the supplement files (Appendix I: EORTC BR-23 Hindi and Marathi). Hence, Questions no. 2, 4, 5, and 9 were not re-translated and adopted as it is in BIS. A minor difference in question no. 9 was observed which was “Have you **felt** dissatisfied with your body?” in the BIS and “Have you **been** dissatisfied with your body?” in the BR-23 questionnaire. Since the translations were already available, they were used as it is in the final version of the BIS translation.

**Fig. 1:**
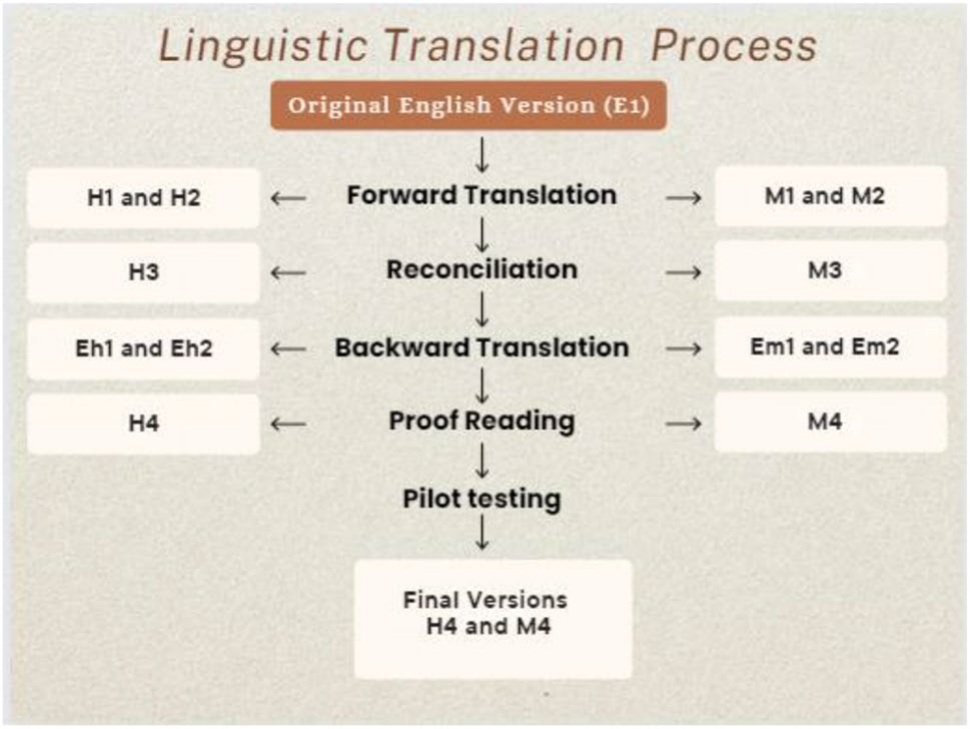
Linguistic translation process

The remaining 6 questions of BIS were taken up for translation so that the entire BIS tool is available for future use in different clinical and research settings. The process of translation of BIS was done using the steps shown in Fig. 1.

### Forward Translation

Two translators each for Hindi and Marathi, who were native speakers of Hindi and Marathi respectively, with good command of the English language, were asked to translate the questionnaire simultaneously into the target language, using easy-to-understand phrases, and attempt to accurately represent the original BIS scale. The translations thus generated were called H1 and H2 and M1 and M2 (2 translations each of Hindi and Marathi, respectively). The cultural differences were duly considered in this process.

### Reconciliation

An independent third translator who is a senior breast radiation oncologist, with a good command of the English language and fluent in both Hindi and Marathi, reviewed both the translations, assessed the meaning, observed the differences, and created an intermediate version combining both the translations based on relevance and ease of understanding. The third translator also coordinated with the previous translators regarding any inconsistencies for reconciliation. The versions thus generated were called H3 and M3 for Hindi and Marathi, respectively.

### Back Translation

The reconciled Hindi and Marathi versions were back-translated into the English language by two independent translators each for Hindi and Marathi (a total of 4 translators), to ensure that the translated versions were similar to the original BIS scale. These translators were senior registrars from Tata Memorial Centre, Mumbai. They were not given the original English scale for reference. Thus, Eh1 and Eh2 and Em1 and Em2 respectively were generated.

### Proofreading

E1 (original), H3/M3 (reconciled versions), and Eh1, Eh2/Em1, Em2 (back-translated versions) were given to a language expert (expert in English and Target language, either Hindi or Marathi) for review. These are available as appendix II in the supplement files. After due discussion, final versions, H4 and M4 were generated. These were considered the best possible translations after ensuring accuracy, relevance, and respecting cultural differences.

### Pilot Testing

A total of 20 breast cancer patients (10 patients comfortable with Hindi language and 10 patients comfortable with Marathi language), undergoing treatment at Tata Memorial Centre, Mumbai, were approached for the pilot testing of the translated versions. They were given the translated version in the respective language and were asked to fill it. Following that, they were interviewed by the team, wherein they were asked to answer either yes or no to the following 4 parameters for all 10 items on the BIS:

1. Difficult to answer
2. Confusing
3. Difficult to understand
4. Upsetting/offensive

Following this, they were interviewed by the team regarding the appropriateness of the translated version and the relevance with their condition. Their comments were recorded. A professional translator was consulted regarding the questions that had less than 80% acceptability in any of the above 4 parameters. Their suggestions were discussed with the patients and the translation team, and final versions were generated after discussion.

## Results

The sequence of questions from the original English version was maintained in both Hindi and Marathi translations. No new abbreviations were created. All the logos, underlines, question marks, and bold types as in the original English format were followed.

### Forward Translations

The results of Hindi and Marathi Forward translations are explained in Tables 1 and 2, respectively.

**Table 1:** Hindi Forward translation

Q. no	Result and explanation
1	H1 and H2 were similar; hence, H3 was directly generated without any changes
3	One word was different in H1 and H2 (दिखावे in H1 and दिखने in H2). The H2 version (दिखने) was accepted in H3 because it was culturally and conceptually more accurate
6	H1 and H2 were slightly different (H1:यौन रूप, समझती हैं and H2: यौन रूप, महसूस कर रहे हैं). For the H3 version, H2 was adopted
7	H1 and H2 were grossly different. Since H2 was more elaborate, clear, and easier to understand, it was adopted in H3 with a minor change (टालते हैं in H2 to टाला in H3)
8	Since H2 was found to be closely resembling the English version, it was adopted in H3 without any change. (H1: कमी महसूस करते है, H2: संपूर्णता में कमी आई है?)
10	H1 used the phrase “ऑपरेशन के निशान” and H2 used only “निशान,” H2 was adopted in the H3 version as it was more accurate and understandable

**Table 2:** Marathi Forward translation

Q. no	Result and explanation
1	M1 used “आत्म-जागरुक,” and M2 used only “जागरुक”. In M3, “आत्म-जागरुक” was finally adopted
3	M1 and M2 were combined to generate M3 due to minor variation in the phrasing of the sentence (M1: समाधानी and M2: असंतुष्ट)
6	M1 and M2 were similar (Sexually attracted was translated to लैंगिक दृष्टी in both M1 and M2). “झाल्यासारखे” was used only in M2 which was finally adopted in M3
7	“आपल्या दिसण्याबद्दल आपणास जसे वाटले” was used in M2 and was adopted in M3, M1 was much shorter and not sufficiently clear
8	M2 was found to be more appropriate, similar to the original; hence, it was adopted in M3 with slight paraphrasing (M1: कमीपणा and M2: संपूर्णता कमी झाल्याचे)
10	“व्रणाच्या दिसण्याबद्दल असमाधानी”, used in M1 was adopted in M3. M2 used “व्रणाबदल असमाधानी,” which was finally rejected and not taken in M3

### Backward Translations

As per the EORTC Manual, backward translation was performed by two independent translators each for Hindi and Marathi, all 4 qualified radiation oncology senior registrars at Tata Memorial Centre Mumbai. The translations are given as appendix II in the supplements file. Overall, both back translations were satisfactory and comparable to E1, with slight paraphrasing. The results and explanation for backward translations for Hindi and Marathi are given in Tables 3 and 4, respectively. For Hindi, the back translations were found to be largely similar to the original questionnaire, with a few discrepancies discussed below. For Marathi, the back translations were very close to E1 with only minor variations, and nothing was found to be grossly different. Most questions used the term “do you feel” instead of “Have you been feeling” used in E1.

**Table 3:** Hindi Backward translation

Q. no	Results and explanation
1	Eh1 used the term “alerted” in place of self-conscious. Eh2 used the phrase “Are you” and “self-aware” instead of “have you been feeling” and “self-conscious” as in the original English version
3	In Eh1, “unsatisfied” and “clothed” were used instead of “dissatisfied” and “dressed,” and the Eh2 version asked the question “are you satisfied?” instead of “are you dissatisfied?”
6	Eh1 used the term “interested” instead of “attractive”. Eh2 was broadly similar to E1
7	Eh1 used the term “ignored” instead of “avoided”, and Eh2 used the term “perceived” instead of “felt”
8	Eh1 used “incompleteness” instead of “less whole”. Similarly, Eh2 also used the term “incomplete”
9	Eh1 was exactly the same as E1 and Eh2 was only slightly different
10	Similar to question 3, “unsatisfied” was used instead of “dissatisfied.” Eh2 was similar to E1

**Table 4:** Marathi Backward translation

Q. no	Results and explanation
1	Em1 used the term “self-aware” instead of “self-conscious”, Em2 was similar to E1
3	Em1 and Em2 were both similar to E1
6	Em1 and Em2 were both similar to E1
7	Both Em1 and Em2 used “perceive” instead of “felt about”. Everything else was broadly similar to E1
8	Both Em1 and Em2 used “incomplete” instead of “less whole”. Everything else was broadly similar to E1
10	Em1 was more concise than E1 asking about “dissatisfaction with scar” instead of “dissatisfaction with the appearance of the scar”. Em2 was only slightly different from E1 due to paraphrasing

### Proofreading

Both translators (Hindi and Marathi) found no major discrepancies, any grossly different meanings, or inappropriate phrases in the translated versions. After discussion with the primary translation team, final versions H4 and M4 were generated for pilot testing.

### Pilot Testing

Ten patients each for Hindi and Marathi, native speakers and comfortable with the respective language, literate (at least secondary level), and undergoing treatment for breast cancer, were administered the questionnaire. After completing the questionnaire, they were interviewed and their comments were recorded. As per the EORTC manual, all patients were asked to answer (yes or no) the following four questions:

1. If the question was difficult to answer
2. If the question was confusing
3. If the question was difficult to understand
4. If it was “upsetting” or “offensive”

Most of the patients found the translated versions easy to understand and clearly framed.

**Fig. 2:**
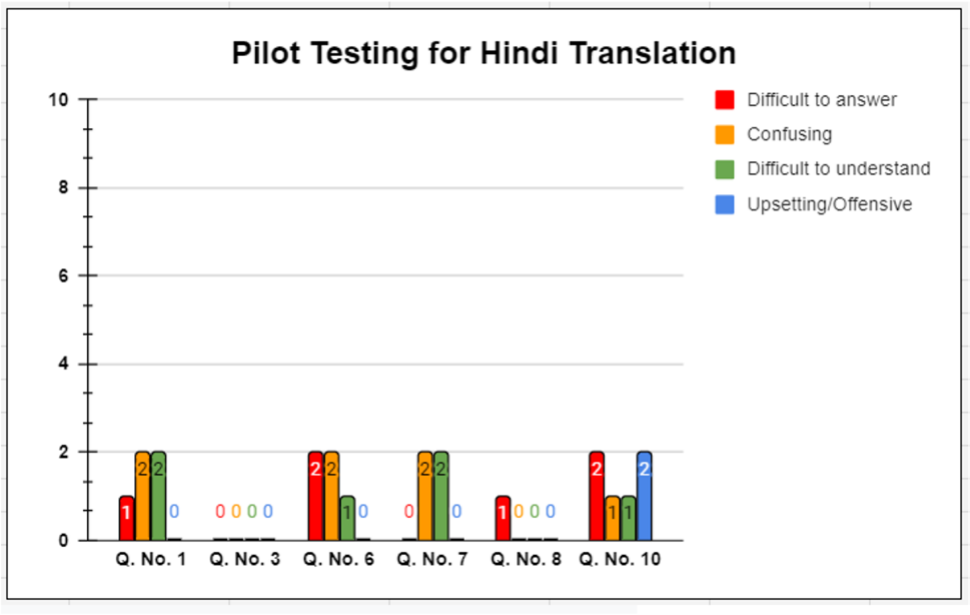
Pilot testing for Hindi translation

**Fig. 3:**
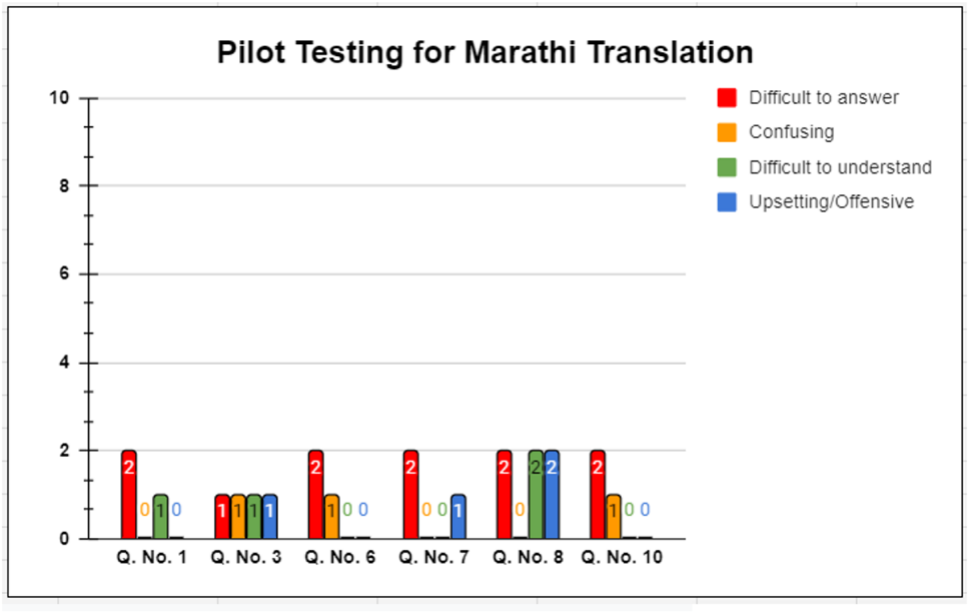
Pilot testing for Marathi translation

For Hindi, all questions had at least 80% acceptability for all 4 parameters, as shown in Fig. 2. Similar results were obtained for the Marathi translation, for all 4 parameters. The results are shown in Fig. 3.

Feedback was taken from professional translator and patients regarding the 20% objections, and due discussion was done with the translation team. Since H4 and M4 were found appropriate and acceptable, they were considered final versions. The final translations (H4 and M4) are available in the supplements file (Appendix III and IV: BIS translated in Hindi and Marathi respectively).

## Discussion

The body image scale is a 10-item scale that was developed to assess changes in body image in patients diagnosed with cancer on a 4-point scale. It was developed by Hopwood et al. in collaboration with EORTC and tested on British cancer patients, for evaluation of psychometric properties. The scale showed high reliability and validity, after which it was found suitable for use in clinical trials [16].

The scale was systematically designed to be suitable for use in cancer patients likely to have body image concerns. The main use was to make comparisons between patient groups. As per Hopwood et al., the minimum no. of items required for such a scale for measurement is 10, which is based on the results of their testing in breast cancer patients. Even though it was tested in breast cancer patients, this scale was designed with a structure that would be applicable across disease sites and treatment situations [16].

The EORTC breast cancer module was developed to address the lack of a suitable scale to measure body image in cancer patients, among various other domains. The EORTC intended to develop a patient-reported outcome measure to be used in conjunction with QLQ-C30 in clinical trials or studies where body image was an important outcome [20]. The four items from the full BIS were incorporated into this breast cancer module (EORTC OLQ-BR23). This was done on the basis of their content validity [19].

The BIS has been translated and validated in various national and international languages. The Spanish version was developed by Gomez-Campelo et al. for use in patients with breast and gynecological cancer patients [21]. The Japanese version of BIS was validated by Sato et al. for bladder cancer [22]. Similarly, the Portuguese version of BIS was analyzed for its psychometric properties for use in breast cancer patients by Moreira et al. [23].

The measurement properties of the BIS in cancer patients were systematically reviewed by Melissant et al., and they found that the BIS had good structural validity, internal consistency, and test–retest reliability [24].

There are limited translation and validation studies available in Indian Languages. The BIS was translated from English to three Indian languages (Tamil Telugu and Hindi) by Shunmugasundaram et al. in 2021 and was later evaluated for psychometric properties for use in head and neck patients [17, 18].

For this report, we chose Hindi because Hindi is a national language which is commonly written and spoken across the country. It is easily understood by majority of the Indian population. Moreover, being a tertiary cancer center, our institute caters to patients coming from various parts of the country. Marathi was chosen because our institute is located in Mumbai, Maharashtra, and Marathi is the commonest regional language of the state. Also, we could not find any available translation of BIS into Marathi. Thus, ongoing studies in breast cancer at our institute will be able to utilize the BIS in clinical research. Similarly, it will also be accessible to other researchers in the country. The BIS is extensively used for reporting of PROMS in oncology [25–27].

The translation process was strictly carried out according to the EORTC translation manual [14]. In an IEC-approved institutional study (CTRI/2020/01/022871), we plan to validate the translated questionnaire in breast cancer patients receiving radiotherapy at our institute.

Since there is no previous Marathi translation available, the current translation can be utilized after the validation process is complete. A Hindi version of BIS is available as discussed above which has been validated for head and neck patients by Shunmugasundaram et al. Their Hindi translation is slightly different from the current translation. The differences are shown in Table 5. These differences may be due to difference in demography of the target population. Head and neck cancers are common in both males and females whereas breast cancer is predominantly a disease of the females. Also, the geographical location for both translations is different, and hence, a slight change of phrasing of sentences and use of verbs is natural.

**Table 5:** Comparison of BIS translation in the current study with historical Hindi translation

Q. no	Current study	Shunmugasundaram et al
1	क्या आप अपने दिखने को लेकर आत्म जागरूक महसूस कर रहे हैं?	क्या आप अपनी रूप के बारे में आत्म जागरूक महसूस कर रहे हैं
2*	**क्या आपका रोग या उपचार के कारण अपना शरीर काम आकर्षण लगा?**	क्या आपने अपनी बीमारी या उपचार के परिणामस्वरूप शारीररक रूप से कम आकर्षक महसूस किया है?
3	क्या आप कपडे पहनने के बाद अपने दिखने से असंतुष्ट हैं?	क्या आप कपडे पहने हुए अपनी रूप से असंतुष्ट हैं?
4*	**क्या आपका रोग या उपचार के कारण अपना स्त्रीत्व कम लगने लगा?**	क्या आप अपनी बीमारी या उपचार के पररणामस्वरूप कम मर्दाना/ज़नाना महसूस कर रहे हैं?
5*	**क्या आपको खुदको नग्न देखने में तकलीफ हुई?**	क्या आपको खुद को नग्न देखना मुश्किल था?
6	क्या आप अपनी बीमारी या उपचार के परिणामस्वरूप यौन रूप से कम आकर्षक महसूस कर रहे हैं?	क्या आप अपनी बीमारी या उपचार के परिणामस्वरूप कम यौन आकर्षक महसूस कर रहे हैं?
7	आप अपने बारे में जिस तरह से महसूस करते हैं, क्या आपने उसके कारण लोगों को टाला?	क्या आप अपनी रूप के बारे में महसूस करने के कारण लोगों से बचते थे?
8	क्या आपको ऐसा महसूस हो रहा हैं कि उपचार के कारण आपके शरीर की संपूर्णता में कमी आई है?	क्या आप महसूस कर रहे हैं कि उपचार के कारण आपका शरीर पूरा का पूरा नहीं है ?
9*	**क्या आप अपने शरीर से असंतुष्ट हैं?**	क्या आप अपने शरीर से असंतुष्ट महसूस किया है?
10	क्या आप अपने निशान के दिखने को लेकर असंतुष्ट हैं?	क्या आप अपने निशान की उपस्थिति से असंतुष्ट हैं?

*Questions no. 2, 4, 5, and 9 have been adopted from the Hindi EORTC BR 23 questionnaire

The pilot testing done on the final version showed that most of the patients found both the translations acceptable in terms of ease of understanding and acceptability; hence, they will be validated on breast cancer patients in the above-mentioned IEC-approved study. The final translations are available as Appendix III and IV (BIS in Hindi and Marathi respectively) in the supplements file.

## Conclusion

As the Hindi and Marathi versions of the BIS have been translated and showed good acceptability in the pilot testing, we plan to validate them in a large cohort of breast cancer patients. Subsequently, the tools will be available for use by other researchers.

## Supplementary Information

Below is the link to the electronic supplementary material.

Supplementary file1 (PDF 290 KB)

Supplementary file2 (PDF 20.0 KB)

Supplementary file3 (PDF 383 KB)

Supplementary file4 (PDF 415 KB)

## Data Availability

All the data that has been collected and analysed is available with the corresponding author, however it cannot be shared due to its confidential and sensitive nature.
