# Supplementary material for: Translation and Linguistic Validation of BIS (Body Image Scale) for Breast Cancer Patients in India
Source: Indian J Surg Oncol. 2024 Aug 14;16(1):203–10. doi: 10.1007/s13193-024-02037-2 (PMC11920465; doi:10.1007/s13193-024-02037-2)
Supplement: Supplementary file 1 — Supplementary file1 (PDF 290 KB) [file 13193_2024_2037_MOESM1_ESM.pdf]

EORTC QLQ – BR23 ई. ओ. आर. टी.सी बी. आर २३

रोगी कभी बताते है कि उन्हे निम्न लक्षण या कष्ट है । पिछले सप्ताह में आपको किस हद तक यह लक्षण या कष्ट थे यह सूचित करे ।

	पिछले सप्ताह में	बिलकूल नही	थोडासा	थोडा अधिक	बहुत अधिक
३१.	क्या आपका मूँह सूखा रहता था?	१	२	३	४
३२.	क्या आपको खाना या पानी हमेशा से अलग लगता था ?	१	२	३	४
३३.	क्या आपने आँखो में दर्द या जलन महसूस की या आपकी आँखो से पानी आता था ?	१	२	३	४
३४.	क्या आपके बाल गिरते है ?	१	२	३	४
३५.	इस प्रश्न का उत्तर तभी दे अगर आपके बाल गिरे हो - क्या आप अपने बाल गिरने से परेशानी थी ?	१	२	३	४
३६.	क्या आपको बीमार लगा या अपनी तबीयत खराब लगी ?	१	२	३	४
३७.	क्या आपको बहुत गर्मी लगी और चेहरा लाल हो गया ?	१	२	३	४
३८.	क्या आपको सरदर्द होता था ?	१	२	३	४

	पिछले सप्ताह में	बिलकूल नही	थोडासा	थोडा अधिक	बहुत अधिक
३९.	क्या आपको रोग या उपचार के कारण अपना शरीर कम आकर्षक लगा ?	१	२	३	४
४०.	क्या आपको रोग या उपचार के कारण अपना स्त्रीत्व कम लगने लगा है ?	१	२	३	४
४१.	क्या आपको खुदको नग्न देखने में तकलीफ हुअी ?	१	२	३	४
४२.	क्या आप अपने शरीर से असंतुष्ट है ?	१	२	३	४
४३.	क्या आप भविष्य के अपने स्वास्थ के विषय में चिंतीत है ?	१	२	३	४

	पिछले चार सप्ताह में	बिलकूल नही	थोडासा	थोडा अधिक	बहुत अधिक
४४.	आपको शारीरीक संबंधो में कितनी रुची थी ?	१	२	३	४
४५.	आप शारीरीक संबंध किस हद तक रखती थी ? (संभोग या बिना संभोग के)	१	२	३	४
४६.	इस प्रश्न का उत्तर तभी दे अगर आपके शारीरीक संबंध रहे है - शारीरीक संबंधो में आपको कितना आनंद आया ?	१	२	३	४

पिछले सप्ताह में	बिलकूल नही	थोडासा	थोडा अधिक	बहुत अधिक
४७. क्या आपके कंधे में या बाँह में दर्द था ?	१	२	३	४
४८. क्या आपका हात या बाँह सूजे थे ?	१	२	३	४
४९. क्या आपको बाँह उपर उठाने में या घुमाने में कष्ट था ?	१	२	३	४
५०. क्या आपको अपने रोगग्रसित स्तन के भाग में कोई दर्द था ?	१	२	३	४
५१. क्या आपको अपने रोगग्रसित स्तन के भाग में सूजन थी ?	१	२	३	४
५२. क्या आपके रोगग्रसित स्तन का भाग ज्यादा संवेदनशील हो गया था ?	१	२	३	४
५३. क्या आपके रोगग्रसित स्तन के भाग की त्वचा में तकलीफ थी, जैसे कि खुजली, सूखापन या छिलना ?	१	२	३	४

EORTC QLQ - BR23

रुग्ण काही वेळा असे सांगतात, की त्यांना पुढील लक्षणे किंवा समस्या जाणवतात. गेल्या आठवड्यात अशा प्रकारच्या लक्षणांचा किंवा अडचणींचा आपल्याला कितपत अनुभव आला याबद्दलची माहिती द्या.

मागील आठवडयात:	अजिबात नाही	थोडा	बराच	खूपच
31. आपल्या तोंडाला कोरड पडली का?	1	2	3	4
32. खाण्या-पिण्याची चव नेहमीपेक्षा वेगळी वाटली का?	1	2	3	4
33. डोळे दुखले, चुरचुरले अथवा डोळ्यातून पाणी आले का?	1	2	3	4
34. आपले केस गळले का?	1	2	3	4
35. केस गळले असतील तरच खालील प्रश्नाचे उत्तर द्या: केस गळल्यामुळे आपण नाराज झालात का?	1	2	3	4
36. आपल्याला आजारी असल्यासारखे वाटले का?	1	2	3	4
37. आपला चेहरा गरम झाला का?	1	2	3	4
38. आपले डोके दुखले का?	1	2	3	4
39. रोगामुळे अथवा उपचारामुळे आपण शारीरिकदृष्टया कमी आकर्षक झाला आहात असे वाटले का?	1	2	3	4
40. रोगामुळे अथवा उपचारामुळे आपले स्त्रीत्व कमी झाल्यासारखे आपणास वाटत आहे का?	1	2	3	4
41. स्वत:ला विवस्त्र बघणे आपल्याला कठीण गेले का?	1	2	3	4
42. आपण आपल्या शरीराबद्दल असमाधानी होता का?	1	2	3	4
43. भविष्यकाळातील आरोग्याबद्दल आपल्याला चिंता वाटली का?	1	2	3	4

मागील <u>चार</u> आठवडयात:	अजिबात नाही	थोडा	बराच	खूपच
44. आपणाला संभोगक्रियेमध्ये कितपत रस वाटला?	1	2	3	4
45. आपण संभोगक्रियेमध्ये कितपत सहभाग घेतला? (समागमासह अथवा समागमाविना)	1	2	3	4
46. आपण संभोगक्रियेमध्ये भाग घेतला असेल तरच या प्रश्नाचे उत्तर द्या.संभोगक्रियेमधून आपल्याला कितपत आनंद मिळाला?	1	2	3	4

कृपया पुढील पानावर जा

मागील आठवडयात:	अजिबात नाही	थोडा	बराच	खूपच
47. आपल्या दंडात किंवा खांद्यात वेदना झाल्या का?	1	2	3	4
48. आपला दंड किंवा हात सुजला होता का?	1	2	3	4
49. आपला दंड वर उचलण्यास अथवा बाजूला हलवण्यास त्रास झाला का?	1	2	3	4
50. रोगपीडित स्तनामध्ये आपणास काही वेदना झाली का?	1	2	3	4
51. रोगपीडित स्तनाला सूज आली होती का?	1	2	3	4
52. रोगपीडित स्तन अतिसंवेदनशील होते का?	1	2	3	4
53. रोगपीडित स्तनावरील अथवा भोवतालच्या त्वचेबद्दल काही तकारी होत्या का (उदा. खाज सुटणे, कोरडी पडणे, पापुद्रा सुटणे)?	1	2	3	4

© Copyright 1994 EORTC Quality of Life Group. सर्व हक्क राखीव. आवृत्ती 1.0
