# Supplementary material for: Translation and Linguistic Validation of BIS (Body Image Scale) for Breast Cancer Patients in India
Source: Indian J Surg Oncol. 2024 Aug 14;16(1):203–10. doi: 10.1007/s13193-024-02037-2 (PMC11920465; doi:10.1007/s13193-024-02037-2)
Supplement: Supplementary file 2 — Supplementary file2 (PDF 20.0 KB) [file 13193_2024_2037_MOESM2_ESM.pdf]

Hindi Back Translation

Q. No.	E1	Eh1	Eh2
1	Have you been feeling self-conscious about your appearance?	Do you feel you have become alerted about your appearance?	Are you self-aware about your external appearance?
3	Have you been dissatisfied with your appearance when dressed?	Are you unsatisfied with your appearance when clothed?	Are you satisfied about your external appearance after getting dressed?
6	Have you been feeling less sexually attractive as a result of your disease or treatment?	Do you feel less sexually interested due to your disease or treatment?	Do you feel sexually less attractive due to your disease or treatment?
7	Did you avoid people because of the way you felt about your appearance?	Have you ignored people because of the way you felt about your appearance?	Do you avoid people because of how you perceive your external appearance?
8	Have you been feeling the treatment has left your body less whole?	Do you have a feeling of incompleteness in your body after treatment?	Do you feel incomplete about your body due to disease or treatment?
10	Have you been dissatisfied with the appearance of your scar?	Are you unsatisfied with the appearance of your surgery scar?	Are you dissatisfied with your scar?

Marathi Back Translation

Q. No.	E1	Em1	Em2
1	Have you been feeling self-conscious about your appearance?	Do you feel you are self-aware about your appearance?	Do you feel self-conscious of your appearance?
3	Have you been dissatisfied with your appearance when dressed?	Do you feel dissatisfied with your appearance when dressed?	Do you feel dissatisfied with your appearance when dressed?
6	Have you been feeling less sexually attractive as a result of your disease or treatment?	Do you feel less sexually attractive due to your disease or treatment?	Do you feel less sexually attractive due to your disease or treatment?
7	Did you avoid people because of the way you felt about your appearance?	Do you avoid people because of how you perceive about your appearance?	Do you avoid people because of how you perceive about your appearance?
8	Have you been feeling the treatment has left your body less whole?	Do you feel your body is incomplete after treatment?	Do you feel your body is incomplete after treatment?
10	Have you been dissatisfied with the appearance of your scar?	Are you dissatisfied with your scar?	Do you feel dissatisfied about your scar appearance?
