# Supplementary material for: Translation and Linguistic Validation of BIS (Body Image Scale) for Breast Cancer Patients in India
Source: Indian J Surg Oncol. 2024 Aug 14;16(1):203–10. doi: 10.1007/s13193-024-02037-2 (PMC11920465; doi:10.1007/s13193-024-02037-2)
Supplement: Supplementary file 3 — Supplementary file3 (PDF 383 KB) [file 13193_2024_2037_MOESM3_ESM.pdf]

बॉडी इमेज स्केल (बीआईएस)

इस प्रश्नावली में आपसे कुछ प्रश्न पूछे जाएँगे जो आपकी बीमारी या उपचार के परिणामस्वरूप आपमें होने वाले बदलाव और आपके दिखने के बारे में आप कैसा महसूस करते हैं उससे संबंधित होंगे । कृपया प्रत्येक प्रश्न को ध्यान से पढ़ें, और उस उत्तर के साथ वाली पंक्ति पर एक टिक लगाएं जो पिछले सप्ताह के दौरान आप अपने बारे में जैसा महसूस कर रहे हैं उसके सबसे करीब हो।

नाम :____________________ दिनांक:____________________

		बिल्कुल नहीं	थोडा सा	थोडा अधिक	बहुत अधिक
१	क्या आप अपने दिखने को लेकर आत्म जागरूक महसूस कर रहे हैं?	____	____	____	____
२	क्या आपका रोग या उपचार के कारण अपना शरीर काम आकर्षण लगा?	____	____	____	____
३	क्या आप कपडे पहनने के बाद अपने दिखने से असंतुष्ट हैं?	____	____	____	____
४	क्या आपका रोग या उपचार के कारण अपना स्त्रीत्व कम लगने लगा?	____	____	____	____
५	क्या आपको खुदको नग्न देखने में तकलीफ हुई?	____	____	____	____
६	क्या आप अपनी बीमारी या उपचार के परिणामस्वरूप यौन रूप से कम आकर्षक महसूस कर रहे हैं?	____	____	____	____
७	आप अपने बारे में जिस तरह से महसूस करते हैं, क्या आपने उसके कारण लोगों को टाला?	____	____	____	____
८	क्या आपको ऐसा महसूस हो रहा हैं कि उपचार के कारण आपके शरीर की संपूर्णता में कमी आई है?	____	____	____	____
९	क्या आप अपने शरीर से असंतुष्ट हैं?	____	____	____	____
१०	क्या आप अपने निशान के दिखने को लेकर असंतुष्ट हैं	____	____	____	____
		लागू नहीं	____		

डॉ. पी. हॉपवुड, सीआरसी मनोवैज्ञनिक दवा समूह, स्टेनली हाउस, क्रिस्टी अस्पताल एन एच एस विश्वास, विल्म्सलो सड़क, विन्गिंगटन, मैनचेस्टर एम20 4बीएक्स दूरभाष: 0161 446 3683 फैक्स: 0161 446 8103
