# Supplementary material for: Translation and Linguistic Validation of BIS (Body Image Scale) for Breast Cancer Patients in India
Source: Indian J Surg Oncol. 2024 Aug 14;16(1):203–10. doi: 10.1007/s13193-024-02037-2 (PMC11920465; doi:10.1007/s13193-024-02037-2)
Supplement: Supplementary file 4 — Supplementary file4 (PDF 415 KB) [file 13193_2024_2037_MOESM4_ESM.pdf]

बॉडी इमेज स्केल (बीआईएस)

या प्रश्नावलीमध्ये तुम्हाला तुमच्या दिसण्याबद्दल आणि तुमच्या आजारामुळे किंवा उपचारांमुळे तुमच्यात होणाऱ्या बदलांबद्दल तुम्हाला कसे वाटते हे विचारले जाईल. कृपया प्रत्येक प्रश्न काळजीपूर्वक वाचा, आणि उत्तराच्या बाजूच्या ओळीवर एक खंबीर चिन्ह लावा जे उत्तर तुम्हाला गेल्या आठवड्यात तुमच्याबद्दल वाटत असलेल्या भावनांच्या अगदी जवळचे असेल.

नाव :________________________ तारीख :________________________

	अजिबात नाही	थोडे	खूप	अत्यधिक
१ आपण आपल्या दिसण्याबद्दल आत्म-जागरुक झाला आहात, असे वाटते का ?	______	______	______	______
२ रोगामुळे अथवा उपचारामुळे आपण शारीरिकदृष्ट्या कमी आकर्षक झाला आहात, असे वाटले का ?	______	______	______	______
३ कपडे परिधान केल्यानंतर आपल्या दिसण्याबद्दल आपण समाधानी होता का?	______	______	______	______
४ रोगामुळे अथवा उपचारामुळे आपले स्त्रीत्व कमी झाल्यासारखे आपणास वाटत आहे ?	______	______	______	______
५ स्वतःला विवस्त्र बघणे आपल्याला कठीण गेले आहे का ?	______	______	______	______
६ रोगामुळे अथवा उपचारामुळे लैंगिकदृष्ट्या कमी आकर्षक झाल्यासारखे आपणास वाटले का ?	______	______	______	______
७ आपल्या दिसण्याबद्दल आपणास जसे वाटले, त्यामुळे आपण लोकांना टाळत होता का ?	______	______	______	______
८ उपचारामुळे तुमच्या शरीराची संपूर्णता कमी झाल्याचे तुम्हाला जाणवत होते का?	______	______	______	______
९ आपण आपल्या शरीराबद्दल असमाधानी होता का ?	______	______	______	______
१० आपण आपल्या व्रणाच्या दिसण्याबद्दल असमाधानी होता का ?	______	______	______	______

लागू नाही__________

डॉ. पी. होपवुड, सीआरसी साइकोलॉजिकल मेडिसिन ग्रुप, स्टेनली हाउस, क्रिस्टी हॉस्पिटल एनएचएस ट्रस्ट, विल्म्सलो रोड, विंटिंगटन, मैनचेस्टर एम२० ४बीएक्स | दूरभाष: ०१६१ ४४६ ३६८३ फैक्स: ०१६१ ४४६ ८१०३ |
